# 5-Fluoro-3-methyl­sulfinyl-2-phenyl-1-benzofuran

**DOI:** 10.1107/S1600536809028189

**Published:** 2009-07-22

**Authors:** Hong Dae Choi, Pil Ja Seo, Byeng Wha Son, Uk Lee

**Affiliations:** a Department of Chemistry, Dongeui University, San 24 Kaya-dong Busanjin-gu, Busan 614-714, Republic of Korea; b Department of Chemistry, Pukyong National University, 599-1 Daeyeon 3-dong, Nam-gu, Busan 608-737, Republic of Korea

## Abstract

In the title compound, C_15_H_11_FO_2_S, the O atom and the methyl group of the methyl­sulfinyl substituent lie on opposite sides of the plane of the benzofuran fragment. The 2-phenyl ring is rotated out of the benzofuran plane, making a dihedral angle of 32.1 (2)°. The crystal structure is stabilized by aromatic π–π inter­actions between the benzene rings of neighbouring mol­ecules [centroid–centroid distance = 3.690 (5) Å]. In addition, the crystal structure exhibits inter­molecular C—H⋯O and C—H⋯F inter­actions.

## Related literature

For the crystal structures of similar 5-halo-3-methyl­sulfinyl-2-phenyl-1-benzofuran derivatives, see: Choi *et al.* (2007*a* ▶,*b* ▶). For the biological and pharmacological activity of benzofuran compounds, see: Howlett *et al.* (1999 ▶); Ward (1997 ▶).
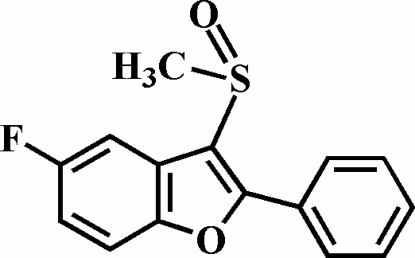


## Experimental

### 

#### Crystal data

C_15_H_11_FO_2_S
*M* *_r_* = 274.30
Monoclinic, 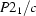

*a* = 8.507 (4) Å
*b* = 16.655 (7) Å
*c* = 9.553 (4) Å
β = 113.732 (5)°
*V* = 1239.1 (9) Å^3^
*Z* = 4
Mo *K*α radiation
μ = 0.27 mm^−1^
*T* = 273 K
0.20 × 0.10 × 0.10 mm

#### Data collection

Bruker SMART CCD diffractometer
Absorption correction: none
8954 measured reflections
2251 independent reflections
1478 reflections with *I* > 2σ(*I*)
*R*_int_ = 0.133

#### Refinement

*R*[*F* ^2^ > 2σ(*F* ^2^)] = 0.061
*wR*(*F* ^2^) = 0.157
*S* = 1.07
2251 reflections
173 parameters
H-atom parameters constrained
Δρ_max_ = 0.64 e Å^−3^
Δρ_min_ = −0.34 e Å^−3^

### 

Data collection: *SMART* (Bruker, 2001 ▶); cell refinement: *SAINT* (Bruker, 2001 ▶); data reduction: *SAINT*; program(s) used to solve structure: *SHELXS97* (Sheldrick, 2008 ▶); program(s) used to refine structure: *SHELXL97* (Sheldrick, 2008 ▶); molecular graphics: *ORTEP-3* (Farrugia, 1997 ▶) and *DIAMOND* (Brandenburg, 1998 ▶); software used to prepare material for publication: *SHELXL97*.

## Supplementary Material

Crystal structure: contains datablocks global, I. DOI: 10.1107/S1600536809028189/er2071sup1.cif

Structure factors: contains datablocks I. DOI: 10.1107/S1600536809028189/er2071Isup2.hkl

Additional supplementary materials:  crystallographic information; 3D view; checkCIF report

## Figures and Tables

**Table 1:** Hydrogen-bond geometry (Å, °)

*D*—H⋯*A*	*D*—H	H⋯*A*	*D*⋯*A*	*D*—H⋯*A*
C5—H5⋯O2^i^	0.93	2.48	3.282 (5)	145
C12—H12⋯O2^ii^	0.93	2.48	3.371 (5)	160
C13—H13⋯O2^iii^	0.93	2.64	3.555 (5)	170
C15—H15*B*⋯O1^iv^	0.96	2.67	3.493 (6)	144
C15—H15*A*⋯F^v^	0.96	2.62	3.509 (6)	155

Symmetry codes: (i) 
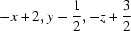
; (ii) 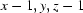; (iii) 
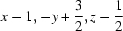
; (iv) 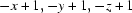; (v) 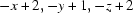.

**Fig. 1.**
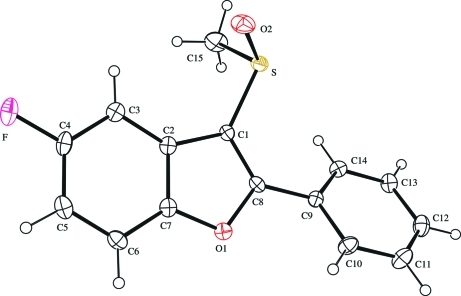
The molecular structure of the title compound with the atom numbering scheme. Displacement ellipsoids are drawn at the 30% probability level. H atoms are presented as a small spheres of arbitrary radius.

**Fig. 2.**
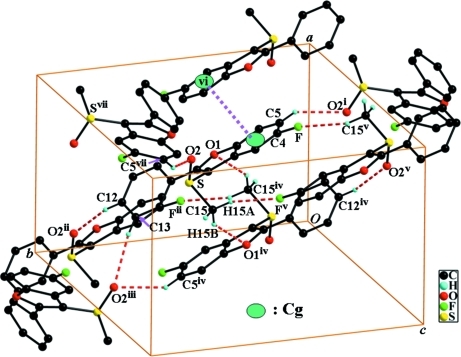
π–π, C–H···O, and C–H···F interactions (dotted lines) in the crystal structure of title compound. Cg denotes the ring centroid. [Symmetry code: (i) -x + 2, y - 1/2, -z + 3/2; (ii) x - 1, y, z - 1; (iii) x - 1, -y + 3/2, z - 1/2 ; (iv) -x + 1, -y + 1, -z + 1; (v) -x + 2, -y + 1, -z + 2; (vi) -x + 2, -y + 1, -z + 1; (vii) -x + 2, y + 1/2, -z + 3/2.]

## supplementary crystallographic information

### Comment

The benzofuran ring systems have been received considerable attenttion in the field of their biological and pharmacological properties (Howlett *et al.*, 1999; Ward, 1997). This work is related to our communications on the synthesis and structures of 5-halo-3-methylsulfinyl-2-phenyl-1-benzofuran analogues, *viz*. 5-bromo-3-methylsulfinyl-2-phenyl-1-benzofuran (Choi *et al.*, 2007*a*) and 5-iodo-3-methylsulfinyl-2-phenyl-1-benzofuran (Choi *et al.*, 2007*b*). Here we report the crystal structure of the title compound, 5-fluoro-3-methylsulfinyl-2-phenyl-1-benzofuran (Fig. 1).

The benzofuran unit is essentially planar, with a mean deviation of 0.011 (3) Å from the least-squares plane defined by the nine constituent atoms. The dihedral angle formed by the planes of the benzofuran and the phenyl rings is 3.690 (5)°. The crystal packing (Fig. 2) is stabilized by aromatic π–π interactions between the benzene rings of the adjacent molecules, with a Cg···Cg distance of 3.690 (5) Å (Cg is the centroid of the C2–C7 benzene ring). The crystal packing (Fig. 2) exhibits four C–H···O and an C–H···F intermolecular interactions (Table 1 and Fig. 2).

### Experimental

The 77% 3-chloroperoxybenzoic acid (291 mg, 1.3 mmol) was added in small portions to a stirred solution of 5-fluoro-3-methylsulfanyl-2-phenyl-1-benzofuran (310 mg, 1.2 mmol) in dichloromethane (30 mL) at 273 K. After being stirred at room temperature for 3h, the mixture was washed with saturated sodium bicarbonate solution and the organic layer was separated, dried over magnesium sulfate, filtered and concentrated in vacuum. The residue was purified by column chromatography (hexane-ethyl acetate, 1 : 1 v/v) to afford the title compound as a colorless solid [yield 83%, m.p. 462-463 K; R_f_ = 0.47 (hexane-ethyl acetate, 1 : 1 v/v)]. Single crystals suitable for X-ray diffraction were prepared by slow evaporation of a solution of the title compound in tetrahydrofuran at room temperature.

### Refinement

All H atoms were positioned geometrically and refined using a riding model, with C–H = 0.93 Å for aromatic H atoms and 0.96 Å for methyl H atoms, respectively, and with *U*iso(H) = 1.2*U*eq(C) for aromatic H atoms and 1.5 *U*eq(C) for methyl H atoms, respectively.

### Crystal data

**Table:**

C_15_H_11_FO_2_S	*F*(000) = 568
*M**_r_* = 274.30	*D*_x_ = 1.470 Mg m^−^^3^
Monoclinic, *P*2_1_/*c*	Mo *K*α radiation, λ = 0.71073 Å
Hall symbol: -P 2ybc	Cell parameters from 2317 reflections
*a* = 8.507 (4) Å	θ = 2.5–26.7°
*b* = 16.655 (7) Å	µ = 0.27 mm^−^^1^
*c* = 9.553 (4) Å	*T* = 273 K
β = 113.732 (5)°	Block, colourless
*V* = 1239.1 (9) Å^3^	0.20 × 0.10 × 0.10 mm
*Z* = 4	

### Data collection

**Table:**

Bruker SMART CCD diffractometer	1478 reflections with *I* > 2σ(*I*)
Radiation source: fine-focus sealed tube	*R*_int_ = 0.133
graphite	θ_max_ = 25.5°, θ_min_ = 2.5°
Detector resolution: 10.0 pixels mm^-1^	*h* = −10→10
φ and ω scans	*k* = −20→20
8954 measured reflections	*l* = −11→11
2251 independent reflections	

### Refinement

**Table:**

Refinement on *F*^2^	Primary atom site location: structure-invariant direct methods
Least-squares matrix: full	Secondary atom site location: difference Fourier map
*R*[*F*^2^ > 2σ(*F*^2^)] = 0.061	Hydrogen site location: difference Fourier map
*wR*(*F*^2^) = 0.157	H-atom parameters constrained
*S* = 1.07	*w* = 1/[σ^2^(*F*_o_^2^) + (0.0649*P*)^2^ + 1.1956*P*] where *P* = (*F*_o_^2^ + 2*F*_c_^2^)/3
2251 reflections	(Δ/σ)_max_ < 0.001
173 parameters	Δρ_max_ = 0.64 e Å^−^^3^
0 restraints	Δρ_min_ = −0.34 e Å^−^^3^

### Special details

**Table:**

Geometry. All esds (except the esd in the dihedral angle between two l.s. planes) are estimated using the full covariance matrix. The cell esds are taken into account individually in the estimation of esds in distances, angles and torsion angles; correlations between esds in cell parameters are only used when they are defined by crystal symmetry. An approximate (isotropic) treatment of cell esds is used for estimating esds involving l.s. planes.
Refinement. Refinement of F^2^ against ALL reflections. The weighted R-factor wR and goodness of fit S are based on F^2^, conventional R-factors R are based on F, with F set to zero for negative F^2^. The threshold expression of F^2^ > 2sigma(F^2^) is used only for calculating R-factors(gt) etc. and is not relevant to the choice of reflections for refinement. R-factors based on F^2^ are statistically about twice as large as those based on F, and R- factors based on ALL data will be even larger.

### Fractional atomic coordinates and isotropic or equivalent isotropic displacement parameters (Å^2^)

**Table:**

	*x*	*y*	*z*	*U*_iso_*/*U*_eq_	
S	0.68313 (13)	0.69115 (6)	0.59163 (12)	0.0254 (3)	
F	1.1429 (3)	0.43327 (15)	0.9193 (3)	0.0410 (7)	
O1	0.6667 (3)	0.49692 (15)	0.3511 (3)	0.0239 (6)	
O2	0.8646 (4)	0.71756 (17)	0.6700 (4)	0.0382 (8)	
C1	0.6897 (5)	0.5933 (2)	0.5247 (4)	0.0195 (8)	
C2	0.8108 (5)	0.5306 (2)	0.6026 (4)	0.0212 (8)	
C3	0.9321 (5)	0.5180 (2)	0.7509 (5)	0.0255 (9)	
H3	0.9490	0.5548	0.8289	0.031*	
C4	1.0255 (5)	0.4483 (3)	0.7757 (5)	0.0280 (9)	
C5	1.0078 (5)	0.3919 (2)	0.6625 (5)	0.0292 (10)	
H5	1.0754	0.3458	0.6864	0.035*	
C6	0.8909 (5)	0.4045 (2)	0.5168 (5)	0.0275 (9)	
H6	0.8777	0.3682	0.4388	0.033*	
C7	0.7926 (5)	0.4737 (2)	0.4896 (4)	0.0224 (8)	
C8	0.6087 (5)	0.5703 (2)	0.3762 (4)	0.0207 (8)	
C9	0.4741 (5)	0.6076 (2)	0.2420 (4)	0.0209 (8)	
C10	0.4709 (5)	0.5957 (3)	0.0963 (5)	0.0303 (10)	
H10	0.5542	0.5640	0.0838	0.036*	
C11	0.3445 (6)	0.6311 (3)	−0.0293 (5)	0.0371 (11)	
H11	0.3438	0.6236	−0.1260	0.044*	
C12	0.2182 (5)	0.6779 (3)	−0.0119 (5)	0.0339 (10)	
H12	0.1329	0.7016	−0.0966	0.041*	
C13	0.2205 (5)	0.6887 (2)	0.1312 (5)	0.0269 (9)	
H13	0.1353	0.7196	0.1428	0.032*	
C14	0.3464 (5)	0.6547 (2)	0.2579 (5)	0.0238 (9)	
H14	0.3467	0.6631	0.3542	0.029*	
C15	0.6122 (6)	0.6672 (3)	0.7391 (5)	0.0403 (12)	
H15A	0.6921	0.6308	0.8104	0.060*	
H15B	0.5009	0.6426	0.6947	0.060*	
H15C	0.6055	0.7155	0.7914	0.060*	

### Atomic displacement parameters (Å^2^)

**Table:**

	*U*^11^	*U*^22^	*U*^33^	*U*^12^	*U*^13^	*U*^23^
S	0.0240 (5)	0.0220 (5)	0.0278 (6)	−0.0001 (4)	0.0079 (4)	−0.0029 (4)
F	0.0295 (14)	0.0525 (17)	0.0367 (15)	0.0104 (11)	0.0086 (12)	0.0157 (12)
O1	0.0211 (14)	0.0263 (15)	0.0259 (15)	0.0005 (11)	0.0112 (12)	−0.0010 (11)
O2	0.0293 (17)	0.0332 (17)	0.048 (2)	−0.0098 (13)	0.0108 (15)	−0.0085 (14)
C1	0.0155 (18)	0.0189 (19)	0.023 (2)	0.0004 (14)	0.0071 (16)	0.0009 (15)
C2	0.0142 (19)	0.026 (2)	0.026 (2)	−0.0018 (15)	0.0110 (17)	0.0027 (15)
C3	0.020 (2)	0.031 (2)	0.027 (2)	−0.0018 (16)	0.0115 (18)	0.0017 (16)
C4	0.016 (2)	0.040 (2)	0.028 (2)	0.0038 (17)	0.0091 (18)	0.0123 (18)
C5	0.027 (2)	0.031 (2)	0.039 (3)	0.0073 (17)	0.022 (2)	0.0095 (18)
C6	0.028 (2)	0.025 (2)	0.036 (2)	0.0015 (17)	0.0193 (19)	−0.0002 (17)
C7	0.020 (2)	0.026 (2)	0.026 (2)	−0.0025 (15)	0.0139 (18)	0.0039 (16)
C8	0.021 (2)	0.0170 (19)	0.028 (2)	0.0004 (15)	0.0135 (17)	0.0001 (15)
C9	0.0177 (19)	0.023 (2)	0.022 (2)	−0.0030 (15)	0.0075 (16)	0.0018 (15)
C10	0.026 (2)	0.040 (2)	0.026 (2)	0.0028 (18)	0.0120 (19)	−0.0037 (18)
C11	0.035 (3)	0.053 (3)	0.025 (2)	0.000 (2)	0.014 (2)	0.000 (2)
C12	0.026 (2)	0.037 (3)	0.034 (3)	−0.0002 (18)	0.007 (2)	0.0096 (18)
C13	0.016 (2)	0.032 (2)	0.034 (2)	0.0027 (16)	0.0114 (18)	0.0044 (18)
C14	0.017 (2)	0.029 (2)	0.026 (2)	−0.0027 (16)	0.0107 (18)	−0.0009 (16)
C15	0.047 (3)	0.043 (3)	0.044 (3)	0.003 (2)	0.033 (2)	−0.008 (2)

### Geometric parameters (Å, °)

**Table:**

S—O2	1.486 (3)	C6—H6	0.9300
S—C1	1.760 (4)	C8—C9	1.470 (5)
S—C15	1.786 (5)	C9—C10	1.395 (5)
F—C4	1.357 (4)	C9—C14	1.397 (5)
O1—C8	1.375 (4)	C10—C11	1.380 (6)
O1—C7	1.381 (5)	C10—H10	0.9300
C1—C8	1.358 (5)	C11—C12	1.390 (6)
C1—C2	1.446 (5)	C11—H11	0.9300
C2—C3	1.392 (5)	C12—C13	1.372 (6)
C2—C7	1.398 (5)	C12—H12	0.9300
C3—C4	1.373 (6)	C13—C14	1.374 (5)
C3—H3	0.9300	C13—H13	0.9300
C4—C5	1.393 (6)	C14—H14	0.9300
C5—C6	1.363 (6)	C15—H15A	0.9600
C5—H5	0.9300	C15—H15B	0.9600
C6—C7	1.385 (5)	C15—H15C	0.9600
			
O2—S—C1	106.19 (17)	C1—C8—C9	133.0 (3)
O2—S—C15	106.3 (2)	O1—C8—C9	115.6 (3)
C1—S—C15	98.6 (2)	C10—C9—C14	119.0 (4)
C8—O1—C7	106.0 (3)	C10—C9—C8	120.4 (3)
C8—C1—C2	106.9 (3)	C14—C9—C8	120.6 (3)
C8—C1—S	124.3 (3)	C11—C10—C9	120.2 (4)
C2—C1—S	127.0 (3)	C11—C10—H10	119.9
C3—C2—C7	119.4 (4)	C9—C10—H10	119.9
C3—C2—C1	135.7 (4)	C10—C11—C12	120.3 (4)
C7—C2—C1	104.9 (3)	C10—C11—H11	119.8
C4—C3—C2	116.3 (4)	C12—C11—H11	119.8
C4—C3—H3	121.9	C13—C12—C11	119.4 (4)
C2—C3—H3	121.9	C13—C12—H12	120.3
F—C4—C3	118.0 (4)	C11—C12—H12	120.3
F—C4—C5	117.8 (4)	C12—C13—C14	121.1 (4)
C3—C4—C5	124.2 (4)	C12—C13—H13	119.5
C6—C5—C4	119.7 (4)	C14—C13—H13	119.5
C6—C5—H5	120.2	C13—C14—C9	120.0 (4)
C4—C5—H5	120.2	C13—C14—H14	120.0
C5—C6—C7	117.2 (4)	C9—C14—H14	120.0
C5—C6—H6	121.4	S—C15—H15A	109.5
C7—C6—H6	121.4	S—C15—H15B	109.5
O1—C7—C6	126.1 (4)	H15A—C15—H15B	109.5
O1—C7—C2	110.6 (3)	S—C15—H15C	109.5
C6—C7—C2	123.3 (4)	H15A—C15—H15C	109.5
C1—C8—O1	111.5 (3)	H15B—C15—H15C	109.5
			
O2—S—C1—C8	−123.7 (3)	C3—C2—C7—C6	−0.5 (6)
C15—S—C1—C8	126.4 (4)	C1—C2—C7—C6	177.9 (3)
O2—S—C1—C2	39.2 (4)	C2—C1—C8—O1	0.4 (4)
C15—S—C1—C2	−70.7 (4)	S—C1—C8—O1	166.2 (3)
C8—C1—C2—C3	178.7 (4)	C2—C1—C8—C9	179.9 (4)
S—C1—C2—C3	13.4 (6)	S—C1—C8—C9	−14.3 (6)
C8—C1—C2—C7	0.6 (4)	C7—O1—C8—C1	−1.3 (4)
S—C1—C2—C7	−164.6 (3)	C7—O1—C8—C9	179.1 (3)
C7—C2—C3—C4	−0.8 (5)	C1—C8—C9—C10	147.8 (4)
C1—C2—C3—C4	−178.6 (4)	O1—C8—C9—C10	−32.7 (5)
C2—C3—C4—F	−178.9 (3)	C1—C8—C9—C14	−32.9 (6)
C2—C3—C4—C5	1.3 (6)	O1—C8—C9—C14	146.6 (3)
F—C4—C5—C6	179.8 (3)	C14—C9—C10—C11	0.8 (6)
C3—C4—C5—C6	−0.4 (6)	C8—C9—C10—C11	−179.8 (4)
C4—C5—C6—C7	−1.0 (6)	C9—C10—C11—C12	−0.8 (7)
C8—O1—C7—C6	−177.6 (4)	C10—C11—C12—C13	0.1 (7)
C8—O1—C7—C2	1.7 (4)	C11—C12—C13—C14	0.6 (6)
C5—C6—C7—O1	−179.3 (3)	C12—C13—C14—C9	−0.6 (6)
C5—C6—C7—C2	1.4 (6)	C10—C9—C14—C13	−0.1 (6)
C3—C2—C7—O1	−179.9 (3)	C8—C9—C14—C13	−179.5 (3)
C1—C2—C7—O1	−1.5 (4)		

### Hydrogen-bond geometry (Å, °)

**Table:**

*D*—H···*A*	*D*—H	H···*A*	*D*···*A*	*D*—H···*A*
C5—H5···O2^i^	0.93	2.48	3.282 (5)	145
C12—H12···O2^ii^	0.93	2.48	3.371 (5)	160
C13—H13···O2^iii^	0.93	2.64	3.555 (5)	170
C15—H15B···O1^iv^	0.96	2.67	3.493 (6)	144
C15—H15A···F^v^	0.96	2.62	3.509 (6)	155

Symmetry codes: (i) −*x*+2, *y*−1/2, −*z*+3/2; (ii) *x*−1, *y*, *z*−1; (iii) *x*−1, −*y*+3/2, *z*−1/2; (iv) −*x*+1, −*y*+1, −*z*+1; (v) −*x*+2, −*y*+1, −*z*+2.
